# Prognosis Following Surgery for Recurrent Ovarian Cancer and Diagnostic Criteria Predictive of Cytoreduction Success: A Systematic Review and Meta-Analysis

**DOI:** 10.3390/diagnostics13223484

**Published:** 2023-11-20

**Authors:** Faiza Gaba, Oleg Blyuss, Dhivya Chandrasekaran, Nicolò Bizzarri, Basel Refky, Desmond Barton, Thomas Ind, Marielle Nobbenhuis, John Butler, Owen Heath, Arjun Jeyarajah, Elly Brockbank, Alexandra Lawrence, Ranjit Manchanda, James Dilley, Saurabh Phadnis, on behalf of the GO SOAR Collaborative

**Affiliations:** 1 Department of Gynaecological Oncology, The Royal Marsden Hospital, London SW3 6JJ, UK; 2 Institute of Applied Health Sciences, University of Aberdeen, Aberdeen AB24 3FX, UK; 3 Wolfson Institute of Preventive Medicine, Barts CRUK Cancer Centre, Queen Mary University of London, London EC1M 6BQ, UK; 4 Department of Pediatrics and Pediatric Infectious Diseases, Institute of Child’s Health, Sechenov University, 119435 Moscow, Russia; 5 Department of Gynaecological Oncology, University College London Hospital, London NW1 2BU, UK; 6 Fondazione Policlinico Universitario A. Gemelli, IRCCS, 00168 Rome, Italy; 7 Department of Surgical Oncology, Mansoura University, El Mansoura 7650030, Egypt; 8 Department of Gynaecological Oncology, The Royal London Hospital, Barts Health NHS Trust, London E1 1FR, UK

**Keywords:** ovarian cancer, recurrence, surgery, prognosis, diagnostics

## Abstract

For women achieving clinical remission after the completion of initial treatment for epithelial ovarian cancer, 80% with advanced-stage disease will develop recurrence. However, the standard treatment of women with recurrent platinum-sensitive diseases remains poorly defined. Secondary (SCS), tertiary (TCS) or quaternary (QCS) cytoreduction surgery for recurrence has been suggested to be associated with increased overall survival (OS). We searched five databases for studies reporting death rate, OS, cytoreduction rates, post-operative morbidity/mortality and diagnostic models predicting complete cytoreduction in a platinum-sensitive disease recurrence setting. Death rates calculated from raw data were pooled based on a random-effects model. Meta-regression/linear regression was performed to explore the role of complete or optimal cytoreduction as a moderator. Pooled death rates were 45%, 51%, 66% for SCS, TCS and QCS, respectively. Median OS for optimal cytoreduction ranged from 16–91, 24–99 and 39–135 months for SCS, TCS and QCS, respectively. Every 10% increase in complete cytoreduction rates at SCS corresponds to a 7% increase in median OS. Complete cytoreduction rates ranged from 9–100%, 35–90% and 33–100% for SCS, TCS and QCS, respectively. Major post-operative thirty-day morbidity was reported to range from 0–47%, 13–33% and 15–29% for SCS, TCS and QCS, respectively. Thirty-day post-operative mortality was 0–6%, 0–3% and 0–2% for SCS, TCS and QCS, respectively. There were two externally validated diagnostic models predicting complete cytoreduction at SCS, but none for TCS and QCS. In conclusion, our data confirm that maximal effort higher order cytoreductive surgery resulting in complete cytoreduction can improve survival.

## 1. Introduction

Advances in ovarian cancer chemotherapeutics and ultra-radical surgery have led to a growing number of long-term survivors. In 1975, the 5-year survival rates were 34% in comparison to 51% in 2015 [1]. 80% with FIGO stage III–IV disease will develop recurrent disease despite primary cytoreduction surgery followed by platinum and taxane-based chemotherapy [1]. At present, there is no standard of care for the management of recurrence. Whilst treatment for recurrent ovarian cancer usually requires multiple lines of systemic therapy, additional cytoreduction surgery may be beneficial. 

The principles used to explain the survival benefit of primary cytoreduction surgery are also thought to apply to recurrent cytoreduction and are believed to be related to theories of tumour cell kinetics and the development of drug resistance. The Gompertz cell growth curve model shows an increased growth rate in the earlier part of the curve when tumours are relatively small [2]; therefore, the log-kill of tumours by chemotherapy is greater in small-volume tumours made up of rapidly growing and dividing cells. Cytoreduction surgery works by removing large tumours with a relatively small growth fraction and leaving behind smaller (or microscopic) tumours with a relatively greater growth fraction (higher proportion of actively dividing cells), making them more susceptible to the effects of cytotoxic chemotherapy. Also, a reduction in the tumour size decreases the adverse metabolic effects of the tumour, leading to symptom relief and improved performance status. Moreover, tumour debulking may enhance tumour perfusion, resulting in improved drug delivery to the target tissues. Another important concept is that decreasing the number of viable tumour cells decreases the rate of somatic mutations that often perpetuate drug resistance [3]. Hence, cytoreduction surgery removes existing resistant tumour cells and decreases the spontaneous development of resistant cells.

Randomised trials have generated conflicting data, exacerbating the controversy of secondary cytoreduction surgery [4,5,6], and there remains a paucity of data on the role of tertiary and quaternary surgery. Therefore, this systematic review and meta-analysis aims to present data in relation to prognosis following recurrent cytoreduction surgery, and diagnostic criteria predictive of complete cytoreduction, to inform patient selection and aid in counselling in this setting of ovarian cancer recurrence.

## 2. Materials and Methods

### 2.1. Search Strategy and Selection Criteria

This systematic review and meta-analysis was performed in accordance with the Meta-analysis Of Observational Studies in Epidemiology (MOOSE) and Preferred Re-porting Items for Systematic Reviews and Meta-Analyses (PRISMA) guidelines. Details of the protocol were registered prospectively on the international PROSPERO database. Five databases were searched from inception to August 2023 using a common search strategy (Supplementary Table S1): Medline, Embase, PubMed, Prospero, Cochrane. Additionally, web-based platforms were searched, including specialised journals, Google searches for grey literature, conference proceedings and clinical trial registries (ISRCTN-registry/ClinicalTrials.gov registry). Searches were not restricted by geographical location, publication year or study design, but were limited to human studies and the English language. The search was re-run prior to final analyses to capture recently published studies.

Predefined inclusion criteria were women >18 years old with platinum-sensitive recurrent epithelial ovarian cancer undergoing recurrence cytoreduction surgery. We excluded women undergoing primary or delayed cytoreduction surgery following initial diagnosis; recurrence surgery for non-epithelial ovarian cancer or platinum-resistant disease; duplicated studies; and studies with abstracts alone with no full text. Outcome measures included death rate (the number of deaths divided by the number of patients in the cohort), median overall survival (OS, defined from date of diagnosis to date of death), complete cytoreduction rate (no macroscopic residual disease), thirty-day post-operative major morbidity (Clavien-Dindo grades III–IV) and mortality, and diagnostic criteria predictive of achieving complete cytoreduction. 

### 2.2. Data Extraction, Quality Assessment and Analysis

Data were extracted using a standardised, predesigned formatted sheet (following piloting and refinement) in Microsoft Excel 2013 by two independent investigators (FG and DC). Inter-rater reliability was analysed using quantity (Q) and allocation (A) disagreements [7]. Any discrepancies were referred to investigators EB and SP for discussion and consensus. Three main categories of data were extracted: methodological characteristics, interventions (secondary (SCS), tertiary (TCS) and quaternary (QCS) cytoreduction surgery defined as cytoreduction surgery after first, second and third recurrence, respectively) and reported outcome measures. The risk of bias was assessed using the Newcastle Ottawa Scale (NOS) [8]. GRADE (Grading of Recommendations Assessment Development and Evaluations) was used to assess the overall quality of evidence for each outcome. No studies were excluded from data synthesis based on quality assessment scores. We tabulated the characteristics and reported outcome measures of all studies for qualitative synthesis. In instances where two or more studies had overlapping datasets, the study with the least risk of bias or highest quality was used for pooling. The decision to conduct a meta-analysis (quantitative data synthesis) was made a posteriori to ensure sufficient studies with similar characteristics were available. As studies varied in their outcome measures, to ensure comparability between studies, the death rate was calculated using raw data independently extracted by authors FG and DC. The authors of studies in which raw data were missing from the published manuscript were contacted.

Since studies differed by year, geographical location, confounders and reported measurements of effect size, the death rate and 95% confidence intervals calculated from raw data were pooled based on a random effects model. The Der Simonian Laird estimate was used to assess study variance. To determine the extent of inter-study variations, we conducted heterogeneity tests with Higgins’ I^2^ statistic to measure the proportion of the observed variance that reflects true effect sizes [9]. An I^2^ ≥ 50% was considered to represent a significant inter-study variation [10]. Meta-regression analysis for the death rate was performed to further explore the role of complete or optimal cytoreduction as a moderator in univariable and multivariable models. Additionally, simple and multiple linear regression analyses were conducted to investigate the association between log-transformed median OS time and the proportion of complete cytoreduction, the proportion of optimal cytoreduction and other clinically important covariates (e.g., age, disease-free interval, post-operative morbidity). A two-sided p-value of less than 0.05 was considered statistically significant, and all statistical analyses were conducted using R version 3.5.1.

## 3. Results

Supplementary Figure S1 provides the flow chart outlining the search outcomes and the study selection process. Searches of electronic databases and reference lists generated 623 references. Upon evaluation of the titles and abstracts, 100 articles were potentially eligible for detailed assessment, of which 76 met our inclusion criteria for qualitative synthesis (Supplementary Table S2). There were high levels of agreement between the reviewers (Q = 1/76, A = 2/76). A total of 64 studies [4,5,6,11,12,13,14,15,16,17,18,19,20,21,22,23,24,25,26,27,28,29,30,31,32,33,34,35,36,37,38,39,40,41,42,43,44,45,46,47,48,49,50,51,52,53,54,55,56,57,58,59,60,61,62,63,64,65,66,67,68,69,70,71] (4 randomised controlled trials, 11 prospective cohort studies and 49 retrospective cohort studies) pertained to SCS, 8 [72,73,74,75,76,77,78,79] (all retrospective cohort studies) to TCS and 4 [80,81,82,83] (all retrospective cohort studies) to QCS. The mean follow-up ranged from 16–88, 13–35 and 18 months for SCS, TCS and QCS, respectively; the median age ranged from 51–64, 51–58 and 54–61 years for SCS, TCS and QCS, respectively. The median OS was reported as 16–91 months for SCS. For patients who had achieved optimal cytoreduction, OS ranged from 24–99 and 39–135 months for TCS and QCS, respectively, while for those who had achieved suboptimal cytoreduction, the OS range was 6–79 and 10–13 months for TCS and QCS, respectively. The definition for optimal cytoreduction varied across studies from no macroscopic disease to <2.5 cm of disease. A range of 14–73% of women undergoing SCS had solitary sites of disease, compared with 17–91% in TCS. One of the four studies reporting QCS reported the proportion of patients with a solitary site of disease (45%). Complete cytoreduction (no macroscopic disease) rates ranged from 9–100%, 35–90% and 33–100% for SCS, TCS and QCS, respectively. The major post-operative thirty-day morbidity was reported to range from 0–47%, 13–33% and 15–29% for SCS, TCS and QCS, respectively. Thirty-day post-operative mortality was 0–6%, 0–3% and 0–2% for SCS, TCS and QCS, respectively. The disease-free interval (DFI, time after the primary treatment until first recurrence) was 10–43 months for SCS, while the treatment free interval (TFI, time without any treatment after recurrent cytoreduction surgery until second/third recurrence) was 4–22 and 14–20 months for TCS and QCS, respectively. Table 1 summarises the diagnostic criteria reported as predictors for achieving complete cytoreduction.

Supplementary Table S3 summarises the risk of bias assessment and Supplementary Table S4 the GRADE assessment for certainty of evidence per outcome. The GRADE certainty for evidence for SCS was high, and moderate for TCS and QCS. According to GRADE, all observational studies have an initial low level of evidence. For SCS, the certainty of evidence was upgraded due to large and consistent size effects and randomised controlled trial data. For TCS and QCS, the certainty of evidence was downgraded due to serious risks of bias and no prospective data, but was upgraded considering the consistent size effect.

**Figure 1:**
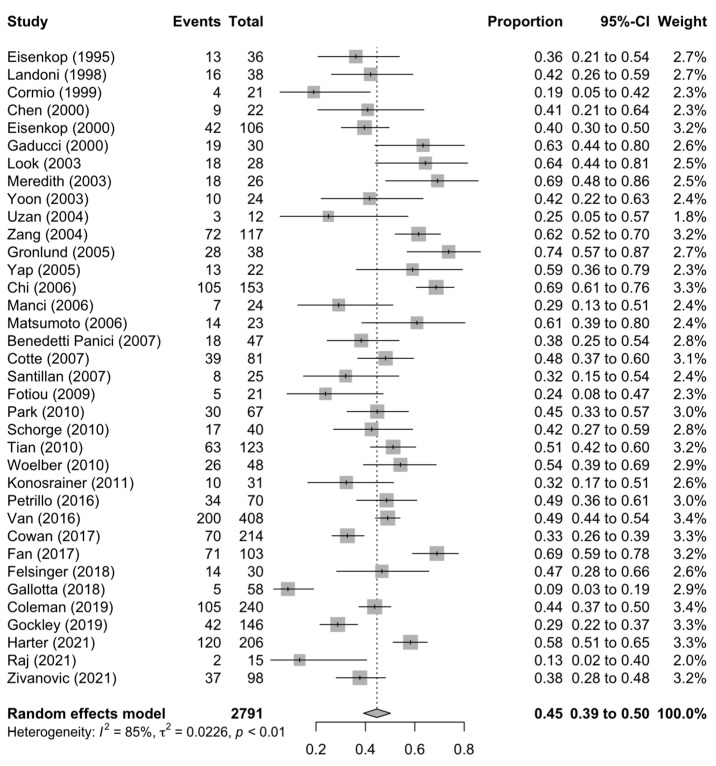
Forest plot of the death rate for secondary cytoreduction surgery [4,6,11,12,13,14,15,16,17,18,19,20,21,22,23,24,25,26,27,29,30,31,32,33,34,35,37,38,39,40,41,42,43,71].

**Figure 2:**
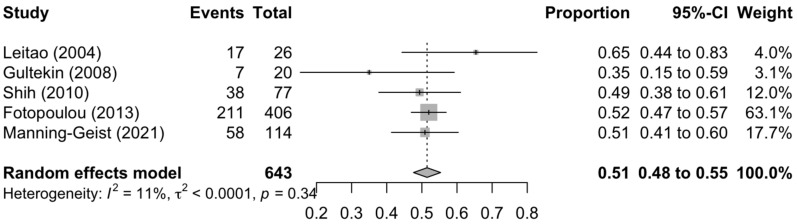
Forest plot of the death rate for tertiary cytoreduction surgery [72,73,76,78,79].

**Figure 3:**
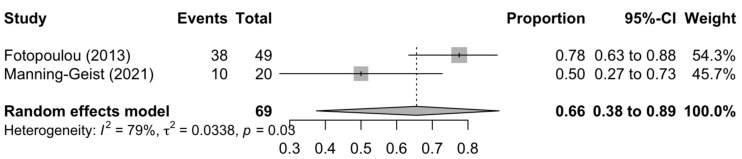
Forest plot of the death rate for quaternary cytoreduction surgery [80,83].

Figure 1, Figure 2 and Figure 3 summarise the death rate for each study pertaining to SCS, TCS and QCS. For SCS, the pooled death rate from 36 studies of 2791 patients was 0.45 (95%CI 0.39–0.50). For TCS, the pooled death rate from five studies of 643 patients was 0.51 (95%CI 0.48–0.55). For QCS, the pooled death rate from two studies of 69 patients was 0.66 (95%CI 0.38–0.89).

The heterogeneity of the models as measured by I^2^ ranged from 11–85%. The SCS and QCS models had high heterogeneity (I^2^ ≥ 50%), while the TCS model had low heterogeneity (I^2^ < 50%). For SCS, fourteen studies were located outside the funnel plot, but these were equally distributed on both sides of the plot, indicating low publication bias (Supplementary Figure S2). It was not possible to assess publication bias for TCS and QCS due to a paucity of studies (<10).

A univariate meta-regression analysis of the death rate for SCS conducted to determine the cause of model heterogeneity showed a statistically significant proportion of complete and optimal cytoreduction (Table 2). Age, DFI and pattern of recurrence and post-operative major morbidity had no impact on the death rate. In the multivariable analysis, complete and optimal cytoreduction remained significant moderators of survival, even after adjusting for age, DFI and major post-operative morbidity. DFI had no impact on the death rate for complete cytoreduction, but a longer DFI was associated with an increase in median OS for optimal cytoreduction. It was not possible to perform a meta-regression for the TCS and QCS models due to a paucity of studies (<10).

The results of the univariable linear regression model for median OS time (Table 3) demonstrated that a higher proportion of complete cytoreduction and a more advanced age were significantly associated with longer OS. Optimal cytoreduction, type of study design (retrospective versus prospective), length of DFI, major post-operative morbidity and patterns of relapse (solitary versus multifocal) did not significantly affect OS. 

The multivariable effect of the proportion of complete and optimal cytoreduction was also evaluated, adjusting for age, DFI and thirty-day post-operative major morbidity. The median OS time increased by 7.43% when the proportion of patients achieving complete cytoreduction increased by 10%, after adjusting for age, DFI and post-operative major morbidity. For complete cytoreduction, for every 1 month increase in DFI, the median OS increased by 2.46%. The change in median OS time was not statistically significant for optimal cytoreduction. Again, it was not possible to perform a linear regression for the TCS and QCS models due to a paucity of studies (<10).

## 4. Discussion

In this systematic review and meta-analysis, we showed a pooled death rate of 45%, 51%, and 66% for SCS, TCS, and QCS, respectively in a platinum-sensitive relapse setting. In addition, results from our meta-regression analysis showed that every 10% increase in complete clearance rates at SCS led to a 7% increase in median OS across all types of study designs. An increase in DFI by 1 month increased the median OS for complete cytoreduction at SCS by 2%. Patterns of relapse (solitary versus multifocal recurrence) failed to significantly affect OS. 

The three RCTs that have published mature data (GOG-213 [4], SOC-1 [5] and DESKTOP III [6]) all demonstrated that patients who have undergone complete cytoreduction at SCS have a significantly longer progression-free survival (PFS) compared with those treated with chemotherapy alone. However, opposing data were generated regarding the impact of SCS on OS. The three-year OS in the tumour-free surgery arm of GOG-213 was 76%, the lowest amongst the three studies (84% DESKTOP III and 78% SOC-1). However, the three-year OS in the non-surgery arm of GOG-213 was the highest at 75% (62% DESKTOP III and 66% in SOC-1). These differences may be due to the lack of standardisation and surgical quality assurance of participating centres, differences in study design and heterogeneous patient profiles between studies. For example, 84% of the study cohort in the GOG-213 received concomitant and maintenance bevacizumab, while it was only used in 23% and 1% of patients in DESKTOP III and SOC-1, respectively, in the non-surgical chemotherapy group. Study data have shown that the use of adjuvant and maintenance bevacizumab among women treated with second-line, platinum-based chemotherapy increases PFS and OS [84,85]. This in part, may explain the similar OS between the surgery and non-surgery arms of the GOG-213 trial. Both the SOC-1 and DESKTOP trials had standardised patient selection criteria for surgery (iMODEL and AGO criteria, respectively), unlike the GOG-213, where patient selection was determined by the surgeon.

In total, we identified eight retrospective studies evaluating TCS and four retrospective studies evaluating QCS. There remains a paucity of high-quality prospective data. However, the reported OS from these studies was similar to that reported by retrospective studies evaluating SCS with comparable short-term morbidity and mortality data (Supplementary Table S2).

Most studies evaluating SCS, TCS and QCS did not incorporate novel targeted agents (anti-angiogenic agents and PARP inhibitors). The GOG-213 study, which included bevacizumab in both the SCS study arm and systemic therapy arm in 84% of the patient sample, showed similar OS. Therefore, the question of whether routine implementation of these novel agents reduces the impact of cytoreduction recurrence surgery remains to be addressed. We would argue that a multimodal personalised collective therapeutic effort involving shared decision-making with the patient, surgeon and oncologist instead of a single treatment modality is vital to optimise survival. For instance, patients with extensive yet operable bowel disease at recurrence would, without surgery, usually not receive bevacizumab due to the risk of perforation. However, they may become eligible by having the bowel disease resected, thereby restoring anatomy and function before commencing systemic treatment. Hence, surgery is able to complement systemic therapy, enabling maximal therapeutic effort to improve survival. The impact of multimodal therapy is indirectly reflected by more recently published studies using anti-angiogenic and targeted therapies alongside surgery, showing improved survival outcomes when compared with older studies [86]. 

Both the iMODEL and AGO risk prediction models have been externally validated in clinical studies to identify patients suitable for achieving complete cytoreduction at SCS. The AGO model was validated in the DESKTOP II trial of 516 patients with a complete cytoreduction rate of 76%. However, the negative predictive value was 38% and the specificity 53% [87]. The iMODEL was externally validated on 117 patients. Complete cytoreduction was achieved in 40% of the cohort, with sensitivity and specificity values of 83.3% and 57.6%, respectively [87]. Limitations of both models include the fact that neither considered surgical ability as an evaluation parameter. Patients with the same prediction scores undergoing SCS performed by different surgeons may obtain different complete cytoreduction rates. They also do not consider BRCA/homologous recombination deficiency (HRD) status or the use of PARP inhibitors and/or bevacizumab. Artificial intelligence models have also been developed to assess the importance of the clinical variables predicting complete cytoreduction at SCS. Three main factors have been proposed to predict complete cytoreduction using artificial neuronal network analysis: DFI (importance = 0.231), retroperitoneal recurrence (importance = 0.178) and residual disease at primary surgical treatment (importance = 0.138) [88]. However, these predictors have not yet been modelled and lack external validity. There are currently no externally validated risk prediction models available to identify patients suitable for achieving complete cytoreduction at TCS and QCS.

The strengths of our systematic review and meta-analysis include a comprehensive search strategy identifying all the relevant literature for inclusion, and methodologically rigorous pooled estimates of death rates following recurrence surgery from raw data resulting in standardised measures of effect sizes from included studies. Overlapping datasets with a greater risk of bias were excluded to ensure no particular dataset was over-represented in our analyses. To limit the risk of reporting bias-influencing findings, all published studies of platinum-sensitive disease recurrence were included. Due to the paucity of studies, meta-regression analysis was not possible for TCS and QCS. There was a large amount of statistical heterogeneity (I^2^ ≥ 50%), and so a random-effects meta-analysis was performed, which produces more conservative confidence intervals. This only partly removed the effects of heterogeneity. Studies across four decades were included in our meta-analysis, resulting in a broad difference in defining optimal residual disease, with residual disease of up to 2.5 cm in older studies considered optimum. Therefore, bulkier disease in the meta-regression analysis might have underestimated the overall survival benefit of <1 cm residual disease. It was not possible to evaluate the impact of hormone receptor status on survival, as this was not reported in the majority of papers included in the meta-analysis.

Our findings are similar to those of previous systematic reviews and meta-analyses evaluating complete cytoreduction at SCS and showing increased OS [86,89]. However, to the best of our knowledge, ours is the first meta-analysis evaluating the death rate for TCS and QCS.

## 5. Conclusions

Multimodal treatment, incorporating both medical chemotherapeutic advances and recurrence surgery, is key to improving survival in a platinum-sensitive recurrence setting. The strongest predictor of survival in all types of recurrence surgery remains the achievement of complete macroscopic cytoreduction irrespective of the number of sites of recurrence and the length of time between relapses. Particularly, for highly symptomatic patients with recurrent disease, surgery provides a much more rapid relief of symptoms compared with systemic therapy options. 

## Figures and Tables

**Table 1:** Diagnostic criteria predictive of achieving complete cytoreduction at recurrence surgery.

Secondary Cytoreduction Surgery	Tertiary Cytoreduction Surgery	Quaternary Cytoreduction Surgery
Platinum sensitive disease	Good performance status (ECOG 0)	≤4 sites of disease
Good performance status (ECOG 0–1)	Age < 60 years	
No residual disease after primary cytoreduction surgery	Size of largest tumour ≤ 8 cm	
FIGO stage I/II disease at diagnosis	Optimal primary (≤1 cm) and secondary (≤0.5 cm) cytoreduction surgery	
Ascites < 500 mls	Time to first recurrence ≤ 32 months	
Serum CA125 at recurrence ≤ 105 U/mL	Time from secondary cytoreduction ≤ 25 months	
Progression free interval ≥ 16 months		

**Table 2:** Meta-regression analysis of the death rate for secondary cytoreduction surgery.

Variable	Estimate	SE	95% CI	z	*p*	No. of Studies	I^2^, %	R^2^, %
**Univariable analysis**								
Proportion of complete cytoreduction	−0.004	0.001	−0.006 to −0.002	−4.025	<0.0001	33	80.7	38.5
Proportion of optimal cytoreduction	−0.005	0.002	−0.008 to −0.002	−3.425	0.0006	33	80.7	30.1
Median age	0.012	0.009	−0.004 to 0.029	1.442	0.149	30	85	1.6
DFI	−0.002	0.005	−0.011 to 0.008	−0.312	0.755	27	86.1	0
Pattern of relapse	0.000	0.002	−0.004 to 0.004	0.016	0.987	18	81.9	0
Post-operative morbidity	−0.003	0.002	−0.008 to 0.001	−1.357	0.175	29	85.4	2.7
**Multivariable analysis**								
**Proportion of complete cytoreduction**	−0.006	0.001	−0.008 to −0.004	−5.322	<0.0001	18	53.9	79
Median age	0.019	0.008	0.003 to 0.036	2.254	0.024			
DFI	0.002	0.005	−0.008 to 0.013	0.449	0.653			
Post-operative morbidity	−0.003	0.002	−0.008 to 0.001	−1.385	0.166			
**Proportion of optimal cytoreduction**	−0.007	0.002	−0.01 to −0.003	−3.739	<0.0001	19	71.2	50.4
Median age	0.014	0.011	−0.007 to 0.035	1.291	0.035			
DFI	0.007	0.005	−0.002 to 0.016	1.563	0.016			
Post-operative morbidity	−0.002	0.003	−0.008 to 0.004	−0.721	0.471			

**Table 3:** Linear regression model for median overall survival time for secondary cytoreduction surgery.

Variable	Beta	SE	t-Value	*p*	Change in Median OS	
					Increase Unit	%
**Univariable analysis**						
Proportion of complete cytoreduction	0.009	0.003	3.433	0.002	10%	9.83
Proportion of optimal cytoreduction	0.008	0.004	1.953	0.062	10%	8.61
Median age	0.043	0.02	2.135	0.044	1 year	4.38
Study design (prospective or retrospective)	0.164	0.13	1.259	0.218	Prospective	17.81
DFI	0.017	0.01	1.658	0.113	1 month	1.71
Pattern of relapse	0.006	0.007	0.904	0.383	10%	6.6%
Post-operative morbidity	−0.001	0.005	−0.1	0.921	10%	−0.1
**Multivariable analysis**						
**Proportion of complete cytoreduction**	0.007	0.002	2.884	0.015	10%	7.43
DFI	0.024	0.01	2.415	0.034	1 month	2.46
Median age	0.006	0.018	0.322	0.754	1 year	0.6
Post-operative morbidity	−0.01	0.005	−1.892	0.085	10%	−9.1
**Proportion of optimal cytoreduction**	0.005	0.004	1.241	0.238	10%	5.63
DFI	0.009	0.013	0.708	0.492	1 month	9.6
Median age	0.028	0.025	1.129	0.281	1 year	32.1
Post-operative morbidity	−0.008	0.008	−1.058	0.311	10%	−7.85

## Data Availability

The datasets used or analysed in this study are publicly available. Data generated from the analysis are presented. Any additional data needed can be made available upon reasonable request from the corresponding author.

## Supplementary Materials

The following supporting information can be downloaded at: https://www.mdpi.com/article/10.3390/diagnostics13223484/s1, Figure S1: PRISMA flow diagram for study selection; Figure S2: Funnel plot of secondary cytoreduction studies for publication bias; Table S1: Search strategy for literature review; Table S2: Qualitative data synthesis of studies reporting the overall survival, cytoreduction rates and 30-day major post-operative morbidity and mortality following recurrence surgery in platinum-sensitive epithelial ovarian carcinoma; Table S3: Risk of bias assessment; Table S4: GRADE assessment of certainty of evidence per outcome.
