# Supplementary material for: Prognosis Following Surgery for Recurrent Ovarian Cancer and Diagnostic Criteria Predictive of Cytoreduction Success: A Systematic Review and Meta-Analysis
Source: Diagnostics (Basel). 2023 Nov 20;13(22):3484. doi: 10.3390/diagnostics13223484 (PMC10670762; doi:10.3390/diagnostics13223484)

Supplementary Figure S1. PRISMA flow diagram for study selection

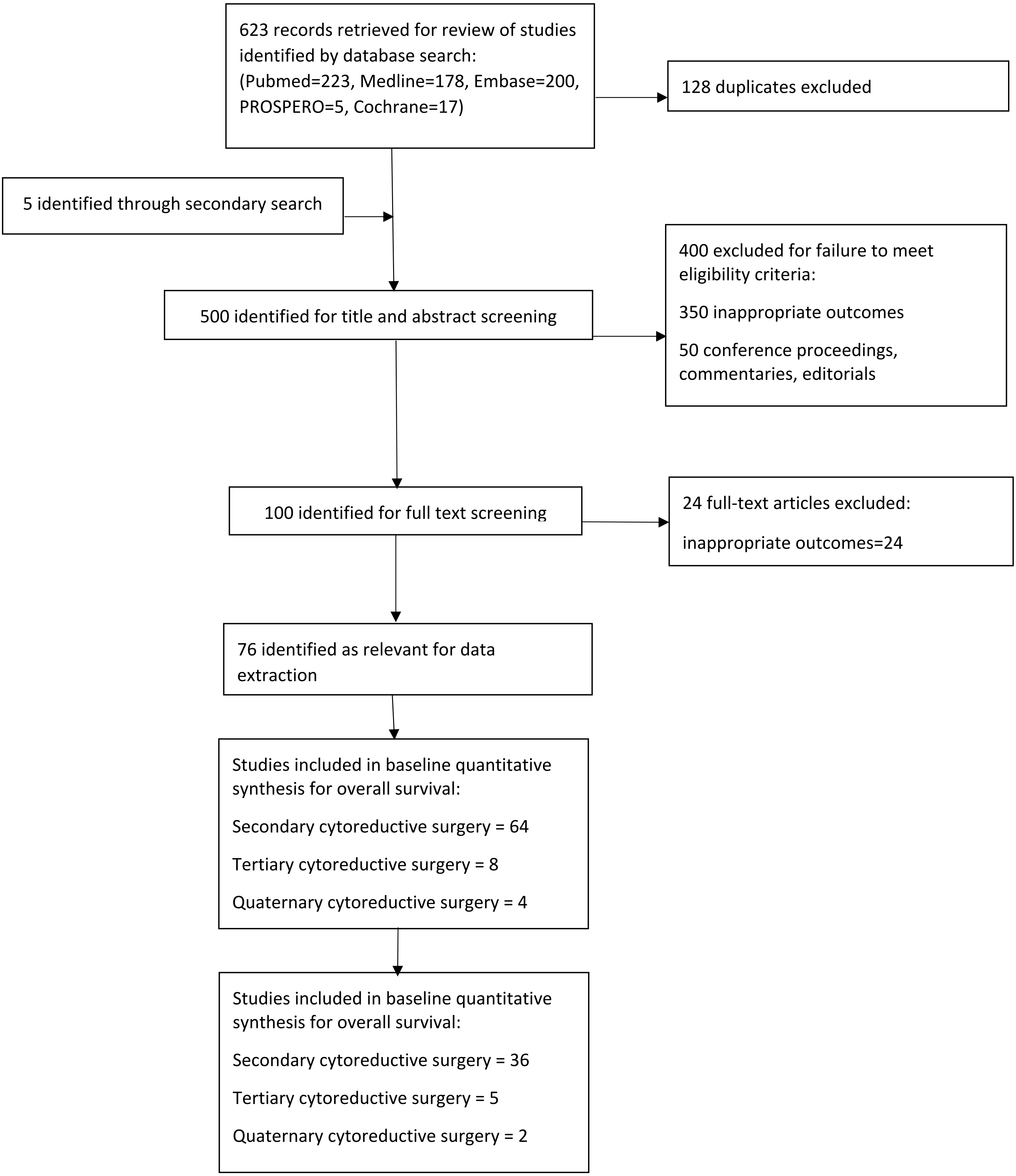

Supplementary Figure S2. Funnel plot of secondary cytoreduction studies for publication bias

.

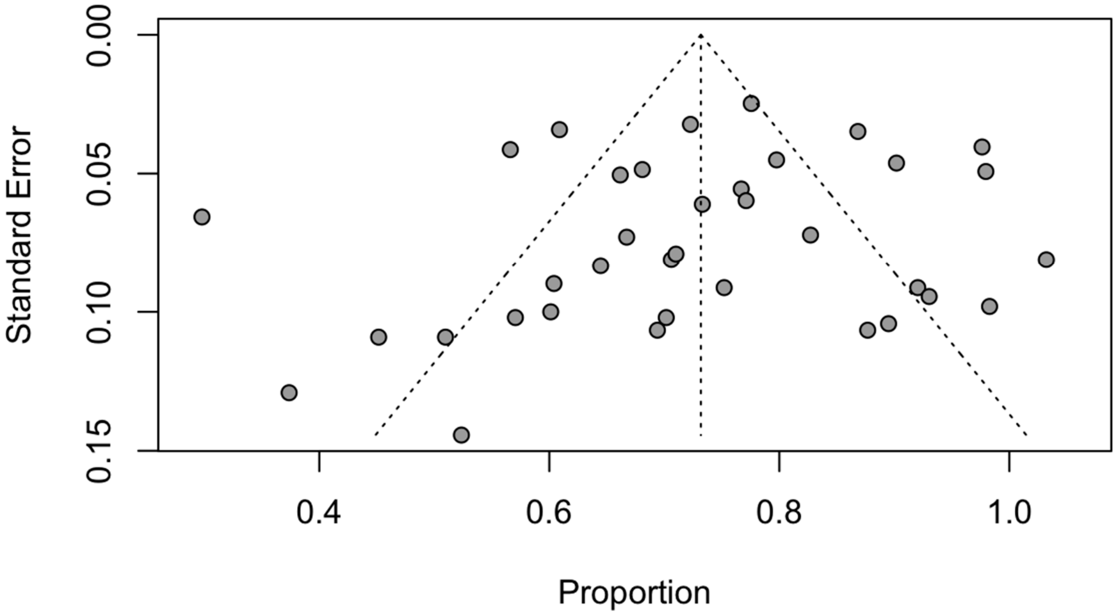

Supplement: Supplementary file 1 [file diagnostics-13-03484-s001.zip › Supplementary_figure_v1.pdf]
