# Supplementary material for: Prognosis Following Surgery for Recurrent Ovarian Cancer and Diagnostic Criteria Predictive of Cytoreduction Success: A Systematic Review and Meta-Analysis
Source: Diagnostics (Basel). 2023 Nov 20;13(22):3484. doi: 10.3390/diagnostics13223484 (PMC10670762; doi:10.3390/diagnostics13223484)
Supplement: Supplementary file 1 [file diagnostics-13-03484-s001.zip › Supplementary_table_v1.pdf]

Supplementary Table S1. Search strategy for literature review

**Eligibility criteria**	Participants: Platinum sensitive epithelial ovarian cancer recurrence<br>Intervention: Recurrent cytoreduction surgery<br>Comparison: No surgery<br>Outcomes: death rate, overall survival, cytoreduction rates, post operative morbidity & mortality
**Data sources**	MEDLINE (1946 to 2023), EMBASE (1974 to 2023), Pubmed (1996 to 2023), PROSPERO (2011 to 2023), Cochrane (1999 to 2023), citation searching, specialist journals, grey literature (conference proceedings/Google search), clinical trial registries (ISRCTN registry/ClinicalTrials.gov registry)
**Search strategy for database searching**	
1. (OVARIAN CANCER).ti,ab	
2. exp "OVARIAN CANCER"/	
3. 1 OR 2	
4. (RECURRENCE).ti,ab	
5. exp "RECURRENCE"/	
6. 4 OR 5	
7. (SURGERY).ti,ab	
8. exp "SURGERY"/	
9. 7 OR 8	
10. (DEATH).ti,ab	
11. exp "DEATH"/	
12. 10 OR 11	
13. (SURVIVAL).ti,ab	
14. exp "SURVIVAL"/	
15. 13 OR 14	
16. exp "CYTOREDUCTION"/	
17. (CYTOREDUCTION).ti,ab	
18. 16 OR 17	
19. (MORBIDITY).ti,ab	
20. exp "MORBIDITY"/	
21. 19 OR 20	
22. (MORTALITY).ti,ab	
23. exp "MORTALITY"/	
24. 22 OR 23	
25. 3 AND 6 AND 9 AND 12 AND 15 AND 18 AND 21 AND 24	

Supplementary Table S2. Qualitative data synthesis of studies reporting overall survival, cytoreduction rates and 30 day major post-operative morbidity and mortality following recurrence surgery in platinum sensitive epithelial ovarian carcinoma

Author	Year	Design	FU (months)	Sample size (N)	Deaths (N)	Mean Age	Median OS	DFI	Solitary metastasis (%)	Optimal cytoreduction criteria	Optimal cytoreduction (%)	Complete cytoreduction (%)	Post operative morbidity (%)	Post operative mortality (%)	Bev/ PARP	HIPEC
**SECONDARY CYTOREDUCTION**																
Morris	1989	Retrospective cohort		30			16.3	42.5		<2	56.7	30	36.7	0		
Janicke	1992	Retrospective cohort		30		53	18	16		<2	86.7	46.7	23.3	0		
Segna	1993	Retrospective cohort		100			16.6			<2	61		13	1		
Eisenkop	1995	Prospective cohort		36	13	60.6	43	22	13.9	≤1	91.7	83.3	30.1	2.8		
Landoni	1998	Retrospective cohort		38	16		29	22		no macroscopic disease	100	100	37	5		
Lichtenegger	1998	Retrospective cohort		63		58	23			<2	65	22	25.9	6		
Cormio	1999	Retrospective cohort	39 (6-96)	21	4	58	29	25		<2		71.4	42.9	0		
Chen	2000	Retrospective cohort	17	22	9	56.5	41	14		<1	86.4	63.6	28.6	0		
Eisenkop	2000	Prospective cohort		106	42	60.5	34.4	16.8	17.9	<0.5	84.9	82.1	32.1	1.9		
Gadducci	2000	Retrospective cohort		30	19	58.5	21	17.5	36.7	<2	83.3	56.7	20	3.3		

Tay	2002	Retrospective cohort	88	46		50.3	22.5	26		≤1	71.7	41.3	10	2.2		
Loizzi	2003	Retrospective cohort	63 (23-250)	31		54	21			<2	96.4					
Look	2003	Prospective cohort	26.9 (0.5-79.1)	28	18	54	45.8			<2.5	75	57.1	10.7	0		**
Meredith	2003	Retrospective cohort	33.2 (3.6-49.6)	26	18	62	26.3			≤1	80.8	69.2	23.1	0		
Yoon	2003	Retrospective cohort	39	24	10	53	62	36.5		≤1	100	87.5	20.8	0		
Uzan	2004	Retrospective cohort	50 (12-158)	12	3	51		21		no macroscopic disease	100	100	25	0		
Zang	2004	Prospective cohort	16 (5-84)	117	72	53	22	15.4	28.2	≤1	61.5	9.4	7.7	0		
Zanon	2004	Prospective cohort	18.9 (2-68.2)	30		60	28.1			≤0.25	76.7		16.7	3.3		**
Gronlund	2005	Retrospective cohort	61.6 (20.1-71.4)	38	28			16.3	31.6	no macroscopic disease	65.8	42.1	5.3	0		
Gungor	2005	Retrospective cohort		44		54.3	16	27.1	63.6	<1		77.3	9	0		
Onda	2005	Retrospective cohort	60 (17-199)	44		52	32	18.5	36.4	<1	84.1	59.1		0		
Yap	2005	Retrospective cohort	24 (6-128)	22	13	57.4	26	48.2	59	<0.5	100		22.7	0		
Ayhan	2006	Retrospective cohort	33.7	64		50.6	18.6	15.5	20.2	≤1	82.8	43.8				

Chi	2006	Retrospective cohort	36.9 (0.16-125.6)	153	105	56.5	41.7	17		≤0.5	51.6	40.5	3.9	0		
Harter	2006	Retrospective cohort	19	267		60	29.2			≤1	75.7	49.8				
Manci	2006	Retrospective cohort	30	24	7	54	56	26	37.5	≤0.5	100		12.5	0		
Matsumoto	2006	Retrospective cohort		23	14	55.7	41.7	22.5	43.5	<2	43.5	30.4	4.3	0		
Park	2006	Retrospective cohort		14		53	32	17		≤0.5	64.3	42.9	35.7	0		
Rufian	2006	Prospective cohort		14		53	57			≤1			14.3	0		**
Benedetti Panici	2007	Prospective cohort		47	18	52	49	15	59.6	≤1	87.2	78.7	19.1	0		
Cotte	2007	Prospective cohort	47.1 (9-203.7)	81	39	54.3	28.4			≤0.5	80.2	55.6	13.6	2.5		**
Salani	2007	Retrospective cohort	30	55		57.7	48			≤1	89.1	74.5	25.5	1.8		
Santillan	2007	Retrospective cohort	19 (7-37)	25	8	59	37	16		≤1	100	96	0	0		
Bae	2009	Retrospective cohort	31 (1-77)	54		54	42	24	22.2	<0.5	87	59.3	20.4	0		
Bristow	2009	Retrospective cohort		56		56	38.4			≤1	92.9	85.7	23.2	1.8		
Cheng	2009	Retrospective cohort		21		53	10			≤1	33					
Fotiou	2009	Retrospective cohort	45	21	5	52	47	21		≤1	90.5	81	0			
Harter	2009	Retrospective cohort		250		60	29.5			≤1	76.4	50	13.6	0		
Park	2010	Retrospective cohort	41 (6-145)	67	30	55	36	20		≤1	61.2	55.2	16.4	0		

Schorge	2010	Retrospective cohort	31 (5-137)	40	17	55.5	54	28	52.5	≤0.5	80	55		2.5		
Tian	2010	Retrospective cohort	26.1 (5.9-80)	123	63	51	31.7	16.1	39.8	≤1	78.9	41.5	4.1	0		
Woelber	2010	Retrospective cohort	13 (1-100)	48	26	60	26	18		<1	47.9	33.3	44	2.1		
Burton	2011	Retrospective cohort		20		59	22.5	18		no macroscopic disease	80	55				
Fagotti	2011	Prospective cohort		41		52.6	38	19		<0.25	100	95.3	35	0		**
Frederick	2011	Retrospective cohort	34.4 (0.8-261.6)	62			71.1	28.2		<1	76.3	36	8.1			
Goto	2011	Retrospective cohort	44 (13-155)	34		57	60	10	65	≤1		74				
Konosrainer	2011	Retrospective cohort	63 (13-170)	31	10	60		25.1		<0.25	90.3	64.5		0		**
Celeen	2012	Prospective cohort	20.8	42		52	37	3				50	21	0		**
Nasu	2014	Retrospective cohort	79.5	16		57.5	48.9	26.5		≤1	95.5	86.4				
Lee	2015	Retrospective cohort	48 (0-68.4)	187		59	49.9			<0.5	92.5	74.9				
Minaguchi	2016	Retrospective cohort	53 (12-195)	80		53.5	46		48.8	<1	86.3	60				
Petrillo	2016	Retrospective cohort		70	34		62		21.4	<0.25	100	88.6	35.7			**
Van	2016	Retrospective cohort	29 (0-168)	408	200	61	51	28	58.1	<0	99.3	72.3	20.1	1.7		

<table>
<tr><td>Cowan</td><td>2017</td><td>Retrospective cohort</td><td>39 (0.4-147.9)</td><td>214</td><td>70</td><td>58.5</td><td colspan="2">82.2</td><td></td><td>42.5</td><td>no macroscopic disease</td><td>86.4</td><td>86.4</td><td></td><td>0</td><td></td><td></td></tr>
<tr><td>Fan</td><td>2017</td><td>Retrospective cohort</td><td>32.1 (2-128)</td><td>103</td><td>71</td><td></td><td colspan="2">60</td><td></td><td>27.2</td><td>≤1</td><td>78.6</td><td>54.3</td><td></td><td></td><td></td><td></td></tr>
<tr><td>Felsinger</td><td>2018</td><td>Retrospective cohort</td><td></td><td>30</td><td>14</td><td>64</td><td colspan="2"></td><td></td><td>73.3</td><td><0.25</td><td>93.3</td><td>80</td><td>0</td><td>0</td><td></td><td></td></tr>
<tr><td>Gallotta</td><td>2018</td><td>Retrospective cohort</td><td>24 (9-71)</td><td>58</td><td>5</td><td>53</td><td colspan="2"></td><td>20</td><td></td><td>no macroscopic disease</td><td>100</td><td>100</td><td>10.3</td><td></td><td></td><td>**</td></tr>
<tr><td>Coleman</td><td>2019</td><td>RCT</td><td></td><td>240</td><td>105</td><td></td><td colspan="2">50.6</td><td>18.9</td><td>36.3</td><td></td><td></td><td>67</td><td>8.9</td><td>0.4</td><td>*</td><td></td></tr>
<tr><td>Gockley</td><td>2019</td><td>Retrospective cohort</td><td></td><td>146</td><td>42</td><td></td><td colspan="2">54</td><td></td><td></td><td><1</td><td>70</td><td>42.5</td><td></td><td></td><td></td><td></td></tr>
<tr><td>So</td><td>2019</td><td>Retrospective cohort</td><td>60.2</td><td>22</td><td></td><td>63.5</td><td colspan="2">91.4</td><td></td><td>2.7</td><td></td><td></td><td>72.7</td><td></td><td></td><td>*</td><td></td></tr>
<tr><td>Harter</td><td>2021</td><td>RCT</td><td>69.8 (59.8-80.4)</td><td>206</td><td>120</td><td>60.8</td><td colspan="2">53.7</td><td>18.4</td><td></td><td>≤1</td><td>79.6</td><td>70.4</td><td></td><td>0</td><td>*
+</td><td></td></tr>
<tr><td>Raj</td><td>2021</td><td>Prospective cohort</td><td></td><td>15</td><td>2</td><td>51</td><td colspan="2">26 (22.9-29)</td><td></td><td></td><td></td><td></td><td></td><td>47</td><td></td><td></td><td>**</td></tr>
<tr><td>Shi</td><td>2021</td><td>RCT</td><td>36 (18-60.6)</td><td>182</td><td></td><td></td><td colspan="2">58.1</td><td>17.4</td><td></td><td></td><td></td><td>76.7</td><td>22.7</td><td>0</td><td>*
+</td><td></td></tr>
<tr><td>Zivanovic</td><td>2021</td><td>RCT</td><td>39.5</td><td>98</td><td>37</td><td>59</td><td colspan="2"></td><td>16</td><td>13.3</td><td>≤0.5</td><td>100</td><td>87.8</td><td></td><td></td><td></td><td>**</td></tr>
<tr><td colspan="18">TERTIARY CYTOREDUCTION</td></tr>
<tr><td rowspan="2">Author</td><td rowspan="2">Year</td><td rowspan="2">Study design</td><td rowspan="2">FU (months)</td><td rowspan="2">Sample size (N)</td><td rowspan="2">Deaths (N)</td><td rowspan="2">Age</td><td colspan="2">Median OS</td><td rowspan="2">TFI from SCR</td><td rowspan="2">Solitary metastasis (%)</td><td rowspan="2">Optimal cytoreduction criteria</td><td rowspan="2">Optimal cytoreduction (%)</td><td rowspan="2">Complete cytoreduction (%)</td><td rowspan="2">Post operative morbidity (%)</td><td rowspan="2">Post operative mortality (%)</td><td rowspan="2">Bev/PARP</td><td rowspan="2">HIPEC</td></tr>
<tr><td>A</td><td>B</td></tr>
</table>

Leitao	2004	Retrospective cohort	22.3 (0-71.7)	26	17	55.5	36.3	10.6	13.4	42.3	≤0.5	73	53.8				
Karam	2007	Retrospective cohort		47		58	24	16	17		<1	81	64	26	0		
Gultekin	2008	Retrospective cohort	15 (1-87)	20	7	51	32	6	4	50	<2	60	35	15	0		
Shih	2010	Retrospective cohort	28.9 (0.7-123.7)	77	38	56.1	55.9	13.6	17	37.7	≤0.5	88.3	72.7	26	0		
Hizli	2012	Retrospective cohort	13 (1-53)	23		58			18	17.4	<1	65.2		13	0		
Fotopoulou	2013	Retrospective cohort	14 (0-182)	406	211	55	49	12					54.1	25.9	3.2		
Fanfani	2015	Retrospective cohort		46			99	79	22	90.6		91.3	77.5				
Manning-Geist	2021	Retrospective cohort	35.4 (0.2-272.9)	114	58	57.5	60.3	27.5	16.1	52.3	≤1	89.5	89.5	33.3	0	* +	
**QUATERNARY CYTOREDUCTION**																	
**Author**	**Year**	**Study design**	**FU (months)**	**Sample size (N)**	**Deaths (N)**	**Age**	**Median OS**		**TFI from TCR**	**Solitary metastasis (%)**	**Optimal cytoreduction criteria**	**Optimal cytoreduction (%)**	**Complete cytoreduction (%)**	**Post operative morbidity (%)**	**Post operative mortality (%)**	**Bev/PARP**	**HIPEC**
							**A**	**B**									
Fotopoulou	2013	Retrospective cohort	18.41	49	38	57	43	13.4			≤0.5	63.2	32.6	28.6	2		
Bacalbașa	2015	Retrospective cohort		20		54.3	55				<1	55	35	15	0		
Fanfani	2015	Retrospective cohort		12			135		20			100	100				
Manning-Geist	2021	Retrospective cohort		20	10	61.1	39.4	9.5	14.1	44.7			85		0	* +	

OS – overall survival (defined as survival from date of recurrence surgery to death); DFI – disease free interval (defined as time after the primary treatment until first recurrence); TFI – treatment free interval (defined as time without any treatment after secondary/tertiary cytoreduction surgery until second/third recurrence); SCR – secondary cytoreductive surgery (defined as cytoreduction surgery after first recurrence of platinum sensitive epithelial ovarian carcinoma); TCR – tertiary cytoreductive surgery (defined as cytoreduction surgery after second recurrence of platinum sensitive epithelial ovarian carcinoma); Bev – bevacizumab; PARP - poly ADP ribose polymerase inhibitor; major post-operative morbidity defined as Clavien Dindo grade III and IV complication.

*Studies reporting use of bevacizumab/olaparib

+ studies reporting use of PARP inhibitor

A optimal cytoreduction

B suboptimal cytoreduction

†overall survival

Supplementary Table S3. Risk of bias assessment

Newcastle Ottawa Score (risk of bias)			
**Studies**	**Selection**	**Comparability**	**Outcome/Exposure**
SECONDARY CYTOREDUCTION			
Morris	Medium (***)	Medium (*)	High (*)
Janicke	Medium (***)	Medium (*)	Medium (**)
Segna	Medium (***)	Medium (*)	Medium (**)
Eisenkop	Medium (***)	Medium (*)	Low (***)
Landoni	Medium (***)	Medium (*)	Low (***)
Lichtenegger	Medium (***)	Medium (*)	Low (***)
Cormio	Medium (***)	Medium (*)	High (*)
Chen	Medium (***)	Medium (*)	High (*)
Eisenkop	Medium (***)	Medium (*)	Medium (**)
Gadducci	Medium (***)	Medium (*)	Medium (**)
Tay	Medium (***)	Medium (*)	Medium (**)
Loizzi	Medium (***)	Medium (*)	Low (***)
Look	Medium (***)	Medium (*)	Medium (**)
Meredith	Medium (***)	High (-)	Medium (**)
Yoon	Medium (***)	High (-)	Medium (**)
Uzan	Medium (***)	Medium (*)	Medium (**)
Zang	Medium (***)	High (-)	Medium (**)
Zanon	Medium (***)	High (-)	Medium (**)
Gronlund	Medium (***)	Medium (*)	Medium (**)
Gungor	Medium (***)	Medium (*)	Low (***)
Onda	Medium (***)	Medium (*)	Medium (**)
Yap	Medium (***)	Medium (*)	Medium (**)

Ayhan	Medium (***)	Medium (*)	Medium (**)
Chi	Medium (***)	Medium (*)	Low (***)
Harter	Medium (***)	High (-)	Medium (**)
Manci	Medium (***)	High (-)	Medium (**)
Matsumoto	Medium (***)	Medium (*)	Low (***)
Park	Medium (***)	Medium (*)	Low (***)
Rufian	Medium (***)	Medium (*)	Low (***)
Benedetti Panici	Medium (***)	Medium (*)	Low (***)
Cotte	Medium (***)	High (-)	Medium (**)
Salani	Medium (***)	High (-)	Medium (**)
Santillan	Medium (***)	Medium (*)	Low (***)
Bae			
Bristow	Medium (***)	Medium (*)	Low (***)
Cheng	Medium (***)	Medium (*)	Low (***)
Fotiou	Medium (***)	Medium (*)	Low (***)
Harter	Medium (***)	High (-)	Medium (**)
Park	Medium (***)	Medium (*)	Medium (**)
Schorge	Medium (***)	Medium (*)	Medium (**)
Tian	Medium (***)	High (-)	Medium (**)
Woelber	Medium (***)	Medium (*)	Medium (**)
Burton	Medium (***)	Medium (*)	Medium (**)
Fagotti	Medium (***)	Medium (*)	Low (***)
Frederick	Medium (***)	Medium (*)	Low (***)
Goto	Medium (***)	Medium (*)	Low (***)
Konosrainer	Medium (***)	Medium (*)	Low (***)

Celeen	Medium (***)	Medium (*)	Medium (**)
Nasu	Medium (***)	Medium (*)	Medium (**)
Lee	Medium (***)	Medium (*)	Medium (**)
Minaguchi	Low (****)	Low (**)	Medium (**)
Petrillo	Medium (***)	Medium (*)	Medium (**)
Van	Medium (***)	Medium (*)	Medium (**)
Cowan	Medium (***)	Low (**)	Medium (**)
Fan	Medium (***)	Medium (*)	Low (***)
Felsinger	Medium (***)	Medium (*)	Medium (**)
Gallotta	Low (****)	Medium (*)	Medium (**)
Coleman	Medium (***)	Medium (*)	Low (***)
Gockley	Medium (***)	Medium (*)	Low (***)
So	Medium (***)	Medium (*)	Low (***)
Harter	Low (****)	Low (**)	Low (***)
Raj	Medium (***)	Medium (*)	Low (***)
Shi	Low (****)	Low (**)	Low (***)
Zivanovic	Low (****)	Low (**)	Low (***)
**TERTIARY CYTOREDUCTION**			
Leitao	Medium (***)	Medium (*)	Medium (**)
Karam	Medium (***)	Medium (*)	Medium (**)
Gultekin	Medium (***)	High (-)	High (-)
Shih	Medium (***)	Medium (*)	Medium (**)
Hizli	Medium (***)	High (-)	Medium (**)
Fotopoulou	High (*)	High (-)	High (*)
Fanfani	Medium (***)	Medium (*)	Medium (**)
Manning-Geist	Medium (***)	Medium (*)	Medium (**)
**QUATERNARY CYTOREDUCTION**			
Fotopoulou	Medium (***)	Medium (*)	Medium (**)
Bacalbașa	High (*)	High (-)	Medium (**)
Fanfani	High (*)	Medium (*)	Medium (**)

Manning-Geist	High (*)	High (-)	High (*)

Low risk of bias: studies that scored four stars for selection, two stars for comparability, and three stars for ascertainment of the outcome/exposure.

Medium risk of bias: studies that scored two to three stars for selection, one for comparability, and two for outcome/exposure ascertainment.

High risk of bias: studies that scored one or zero for selection, zero for comparability, one or zero for outcome/exposure ascertainment.

Supplementary Table S4: GRADE assessment of certainty of evidence per outcome

Outcome	Number of studies	Certainty of evidence (GRADE)
Secondary cytoreduction surgery	64	High*
Tertiary cytoreduction surgery	8	Moderate**
Quaternary cytoreduction surgery	4	Moderate**

GRADE - Grading of Recommendations, Assessment, Development and Evaluations

*Majority of studies were observational studies, which have an initial low level of evidence. Certainty of evidence was upgraded due to large and consistent size effects and presence of randomised control trial data.

**All included studies were observational studies, which have an initial low level of evidence. Certainty of evidence was downgraded due to serious risks of bias. However, certainty of evidence was upgraded taking into account consistent size effect.
